# Unmet Needs in Endometriosis: Lessons from COVID-19

**DOI:** 10.1089/whr.2022.0051

**Published:** 2022-11-14

**Authors:** Niamh Waters, Louis Taffs, Jennifer L. Marino, Charlene Rapsey, Jane E. Girling, Michelle Peate

**Affiliations:** ^1^ Department of Obstetrics and Gynaecology, The University of Melbourne, The Royal Women's Hospital, Parkville, Australia.; ^2^ Department of Paediatrics, The University of Melbourne, Parkville, Australia.; ^3^ Centre for Epidemiology and Biostatistics, The University of Melbourne, Parkville, Australia.; ^4^ Centre for Adolescent Health, Murdoch Children's Research Institute, Parkville, Australia.; ^5^ Department of Psychological Medicine, University of Otago, Dunedin, Aotearoa, New Zealand.; ^6^ Department of Anatomy, School of Biomedical Sciences, University of Otago, Dunedin, Aotearoa New Zealand.

**Keywords:** endometriosis, COVID-19, unmet needs, quality of life, menstruation, qualitative

## Abstract

**Background:**

One key challenge of the COVID-19 pandemic is health care access. Government-imposed restrictions and increased health care burden have induced considerable changes to health care services and their delivery. These are likely to have substantially impacted those with chronic conditions such as endometriosis, as they require sustained management.

**Aims:**

Our objective was to explore the impact of the COVID-19 pandemic on the experience of people with endometriosis, and to use this information to inform health care delivery for the management of chronic conditions in a COVID-normal future.

**Materials and Methods:**

Invitation to participate in an open-ended online survey through social media of Australian endometriosis organizations and the Royal Women's Hospital, Melbourne. Surveys were analyzed qualitatively through template analysis.

**Results:**

Of 576 surveys returned, 329 reported COVID-19 having an impact. Fifteen areas of impact were identified and grouped under three domains: impact on access to health care services, impact on daily life, and impact of isolation. Common impacts included reduced access to health care services, improved symptom management due to decreased day-to-day travel and work-from-home arrangements, and both positive and negative views of telehealth services.

**Conclusions:**

This study provides in-depth insight into the experiences of people with endometriosis during the COVID-19 pandemic, confirming previous studies' findings and offering insight into discrepancies between the Australian Healthcare system categorization of surgeries as “non-essential,” and patient views of these procedures as “essential” to their well-being. Results may inform future adjustments to health care services and delivery to improve the lives of people with endometriosis, and by extension, other chronic conditions.

## Introduction

The coronavirus pandemic has caused substantial changes to health care service delivery, with each country facing challenges in managing COVID-19-related illness in conjunction with routine health care. To reduce community transmission of SARS-CoV-2 in Australia, the government implemented lockdowns (*i.e*., limiting exercise, travel, work, and socializing) and health organizations implemented service changes (*i.e*., postponing all elective surgeries and reducing the number of face-to-face medical and allied health appointments).^1^ Although these policy changes were successful in virus suppression, they decreased access to medical care, increasing the risk of injury, especially for those with chronic health conditions that require sustained management.^2^

These impacts on chronic health can be seen in people with endometriosis. Endometriosis is an inflammatory gynecological disease affecting ∼176 million women worldwide and is defined by the presence of endometrial-like tissue outside the uterus.^3–5^ Endometriosis commonly results in painful menstruation and infertility.^5^ The etiology of endometriosis remains uncertain, and pharmaceutical and surgical therapies have limited success.^3,4,6^ As a result, pain and inflammatory symptoms are often self-managed by patients, adjunct to allied health and alternative care.^7,8^ It is likely, therefore, that the COVID-19-related changes in health service delivery has impacted the lives and health of those living with endometriosis.^9^

This study aimed to explore the patient experience of people with endometriosis during the COVID-19 pandemic, to provide insights into the impact of these changes to health care and health care delivery. These insights into what worked (or did not) for patients can subsequently be applied to ongoing future management of chronic illness.

## Materials and Methods

This article presents the findings of a sub-study of a larger survey of the unmet needs of people with endometriosis. We conducted an anonymous online nonprobabilistic survey of unmet needs in endometriosis, which included 10 open-ended questions asking participants to convey “any wants or needs that related to” multiple aspects of care, including health care professionals, access to care, daily living, employment and education, financial matters, spirituality and religion, social relationships, and information.^10,11^ The last question asked participants about any changes in these needs during the COVID-19 pandemic, the results of which are presented here. The question read—“Due to the COVID-19 pandemic, have any of your needs or wants regarding endometriosis changed? If yes, please describe how these needs and wants have changed.” An inductive response-based analysis approach was taken to derive meaning from participant's experience.

### Setting and sampling strategy

**FIG. 1.**
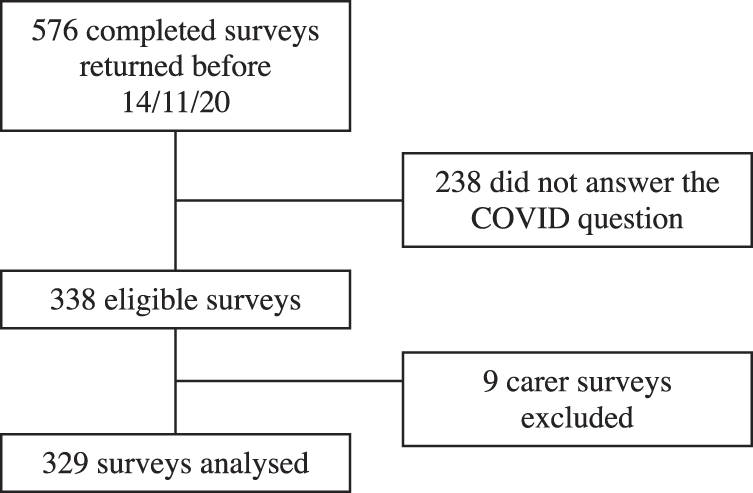
Flowchart of the selection process for surveys included in this analysis.

Participants were invited to complete the survey through social media posts from relevant support and advocacy organizations. Inclusion criteria for this sub-study were aged ≥18 years and a diagnosis of endometriosis from a health care practitioner. Exclusion criteria were being a carer of someone diagnosed with endometriosis, and not responding to the COVID-19 question of the survey (Fig. 1). We used self-reported data on whether participants had been diagnosed with endometriosis by a medical practitioner.

Data were collected between May 24, 2020 and November 14, 2020, 2 months after national restrictions were first introduced. During this time, each state had varying levels of restrictions on nonessential gatherings, in-person workplaces, retail, and services. Interstate travel was very limited. In the state of Victoria (home of 26% of the Australian population^12^), more stringent restrictions were imposed from July 8, 2020 to October 27, 2020. Victorian residents could only leave the house for 1 hour of exercise a day, caregiving, shopping for essential items (*i.e*., groceries and medical supplies), and seeking medical care.

Survey data were collected and stored on the secure web-based software platform REDCap (Research Electronic Data Capture) version 10, hosted at The University of Melbourne.^13^ Electronic consent was required before participants were able to access the survey.

### Analysis

Data were analyzed using Brooks et al.'s method of template analyses^14^ performed in the qualitative analysis software NVivo version 12 (QSR International). Two researchers (N.W. and L.T.) iteratively developed a coding tree through individual analysis of preliminary responses, followed by discussion and consensus with all authors. As new codes were identified, they were added to the coding tree. All responses were double coded with a high level of agreement (>95%). Differences were discussed and adjusted as appropriate. As the mode of data collection did not allow for iterative data collection and analysis, it would be inappropriate to aim for data saturation, thus all responses were coded. Owing to the larger than normal sample size for a qualitative study we have also presented the number of participants per code/theme.

Ethics approval was obtained from the Human Ethics Advisory Group at the University of Melbourne (Ethics ID: 2056522) on April 23, 2020. Informed consent was obtained from participants before being able to access the survey.

## Results

Five hundred seventy-six surveys were returned, 329 (57%) provided responses to the COVID-19 item, and thus were included in this analysis. Participants had a median age of 30 years (range 18–55); the majority (83%) lived in a major city, 46% lived in Victoria, and 68% had a tertiary-level education. Sociodemographic information is summarized in Table 1.

**Table 1.** Participant Demographics (*N* = 329)

Variable	*N* (%)
Age (years)	
Median, IQR, range	30, 25–36, 18–55 (8 missing)
Gender	
Female	324 (98.5)
Male	0 (0.0)
Nonbinary/gender fluid	4 (1.2)
Other^a^	1 (0.3)
Sex assigned at birth	
Female	328 (99.7)
Male	0 (0.0)
Prefer not to say	1 (0.3)
Time since diagnosis	
<1 Year	64 (19.5)
1–5 Years	139 (42.2)
5–10 Years	65 (19.8)
>10 Years	61 (18.5)
Born in Australia	
Yes	295 (89.7)
No	33 (10.0)
Missing	1 (0.3)
Ethnicity	
Anglo-Saxon/English	280 (85.1)
Aboriginal/Torres Strait Islander	5 (1.5)
Chinese	1 (0.3)
Indian	3 (0.9)
Italian	15 (4.6)
Other^b^	40 (12.2)
Languages spoken at home	
English only	310 (94.2)
English and other^c^	12 (3.6)
Other only	0 (0.0)
Missing	7 (2.1)
Highest level of education completed	
Primary school	1 (0.3)
Secondary school	42 (12.7)
TAFE or college	63 (19.1)
Tertiary/postgraduate	223 (67.8)
State	
Victoria	152 (46.2)
New South Wales	59 (17.9)
Queensland	42 (12.8)
Western Australia	28 (8.5)
Australian Capital Territory	20 (6.1)
South Australia	19 (5.8)
Tasmania	6 (1.8)
Northern Territory	1 (0.3)
Missing	2 (0.6)
Remoteness index^d^	
Major cities	272 (82.7)
Inner regional	42 (12.8)
Outer regional	12 (3.6)
Remote	1 (0.3)
Very remote	0 (0.0)
Missing	2 (0.6)

^a^ “My sex is female—gender is a social construct.”

^b^ Other ethnicities listed: Anglo-Asian mix (*n* = 5), Anglo-Nepali (*n* = 1), Anglo-Serbian (*n* = 1), Brazilian (*n* = 1), Brazilian/Australian (*n* = 1), Croatian (*n* = 4), Croatian/Australian (*n* = 1), Dutch and Irish (*n* = 1), German and Hungarian (*n* = 1), Greek (*n* = 2), Irish (*n* = 1), Irish/Australian (*n* = 1), Irish/Scottish/English (*n* = 1), Italian/Australian (*n* = 1), Jewish (*n* = 1), Korean (*n* = 1), Latin (*n* = 1), Lebanese (*n* = 1), Mediterranean (*n* = 1), “my dad is Fijian-Indian and English and my mum is Irish, Spanish and French” (*n* = 1), Pacific Islander (*n* = 1), Polish (*n* = 1), Polish/Australian (*n* = 1), Russian and European mix (*n* = 1), Sri Lankan (*n* = 4), Taiwanese (*n* = 1), Ukrainian (*n* = 1), Vietnamese (*n* = 1), West Indian and Welsh (*n* = 1).

^c^ Languages spoken in addition to English: Auslan, Croatian, German, Greek, Gujarati, Maori, Polish, Portuguese, Sinhala, Spanish.

^d^ For postcodes that returned multiple Accessibility/Remoteness Index of Australia (ARIA) codes, we reported the least remote code.

IQR, interquartile range.

Fifteen themes (areas of impact) were identified and organized under three domains: access to health care services, daily life, and isolation. Domains and their constituent themes are summarized in Table 2. One hundred eighteen participants (36%) reported no impacts of COVID-19. All areas of impact expressed by participants were reported under their corresponding theme, regardless of how many people noted them. There were several points raised by singular participants, all of which were included here to highlight the full range of impacts of the COVID-19 pandemic on the needs of those with endometriosis.

**Table 2.** Domains and Their Constituent Themes, Reflecting the Changes in Unmet Needs Due to the COVID-19 Pandemic for People with Endometriosis

Domain	Theme
Access to health care services	General access to health care
	Telehealth
	Surgery
	Access to allied health
	Access to medication
	Hesitation to seek care
	*In vitro* fertilization (IVF)
Daily life	Support people
	Symptom management
	Work from home
	Financial
Isolation	Shared experience
	Social impact
	Mental health
	Self-reflection

### Domain 1: access to health care services

The domain of access to health care services comprises seven themes: general access to health care, access to allied health, surgery, access to medication, Telehealth, hesitation to seek care, and *in vitro* fertilization (Table 2).

Reduced access to health care services was a primary consequence of the pandemic (*n* = 56). This included canceled or postponed appointments, reduced face-to-face services, and longer wait times to see specialists and gynecologists. Allied health services, such as remedial massages, traditional Chinese medicine (TCM), and physiotherapy were also disrupted, decreasing symptom management and exacerbating pain (*n* = 17). Reduced access negatively impacted both physical and mental health, contributing to worsening of symptoms and feelings of uncertainty.

I have been unable to see my TCM practitioner, no acupuncture, Chinese herbs, pelvic floor therapy. It has also affected my mental health, diet and exercise. Overall it has definitely been a massive change for the worse for my health.—23, major city, diagnosed 1–5 years ago

Cancellation of treatment-related surgeries was reported by 52 participants, and 9 reported postponed fertility treatments. A further nine responses highlighted distress at the language used surrounding surgeries, specifically that the terms “non-essential” or “elective” (used by government and other health bodies) trivialized their experience. Some participants related that this language was consistent with prior dismissal of their health concerns.

… It might not be essential as its not recognised as a debilitating disease. I am left to suffer in pain until such a time its suitable to operate.—32, major city, diagnosed more than 10 years ago

Poor access to doctors limited new prescriptions (*n* = 6). Pandemic-related medication shortages and “panic buying” reduced availability and increased cost (*n* = 8):

I need better stock management for analgesic medication that does not allow bulk buying to result in an inability of people requiring these medications to go without.—21, major city, diagnosed 1–5 years ago

There was also a reluctance to access medical care due to the pandemic (*n* = 19), driven by multiple factors, including fear of being exposed to COVID-19 (*n* = 6), concern about lower quality care (*n* = 5), lower prioritization of health (*n* = 1), or feeling that a doctor's time would be better spent with others (*n* = 1).

I have found myself saying “no you're fine, you don't need to go to hospital or the doctor” because I think it's the safest choice or that I'll just get rushed through, pushed in and out and moved on.—24, major city, diagnosed 1–5 years ago

The restriction of visitors and support people during medical appointments was also distressing (*n* = 6) and generated hesitancy in seeking care (*n* = 1).

Conversely, two participants reported *increased* access to health care services due to reduced wait times and new provision of online yoga and meditation services.

Telehealth services were introduced in response to the pandemic. For 12 participants, this was beneficial, and three wanted services to continue postpandemic. Conversely, 20 participants felt that telehealth consultations were not appropriate for their circumstances and the standard of care was lower than that of in-person appointments. One participant noted that despite this perceived reduction in care, there was no cost reduction.

I still had to pay $145 and the phone call was 4 minutes. It felt rushed and I was left very disheartened. When the appointments are in person, I feel you get better care.—32, inner regional, diagnosed 1–5 years ago.

One hearing-impaired participant indicated that it was more difficult to communicate online than in person.

### Domain 2: daily life

The domain of daily life comprises four themes: support people, symptom management, work from home, and financial (Table 2).

Reduced access to health care services decreased participants' ability to manage their symptoms (*n* = 6). Furthermore, increased stress, changes in daily routine, and government restrictions also contributed to painful flare-ups, fatigue, and poorer mental health (*n* = 13).

Financial issues exacerbated stress, severity of symptoms, and willingness to prioritize health for some (*n* = 12). This further decreased access to health care services, in addition to previously discussed restrictions (*n* = 4).

Sixteen participants reported that their symptom management was aided by work-from-home arrangements. This included the reduction of commutes (*n* = 4), flexible working arrangements (*n* = 4), and greater access to management techniques that participants felt uncomfortable using in their usual workplace (heat packs [*n* = 3] and comfortable clothing [*n* = 2]).

Working from home during COVID has been positive for me, on those days of extreme pain, I have been able to lay down and work or take a bit of time here and there, sit in the sun (I find this helps me). I have been able to do all of this without taking time off. It would be great to have this flexibility all the time.—31, major city, diagnosed 1–5 years ago

Working from home was detrimental to one participant as they were unable to do their job properly, heightening stress and symptoms.

### Domain 3: isolation

The domain of isolation comprises three themes: social impact, mental health, and self-reflection (Table 2).

Feelings of social isolation were worsened by COVID-19 restrictions as participants were unable to give and receive support from others (*n* = 7). The social isolation, uncertainty, and disruption to daily life due to COVID-19 was not dissimilar to the regular experiences of those with endometriosis (*n* = 3). This was seen to be a positive outcome of the pandemic as the general public gained a better understanding of the everyday experience of those with chronic conditions (*n* = 1):

People with chronic illnesses miss out on social gatherings and events more than most, we are acclimatised to self-isolation. The wider community is complaining about being locked up in their houses without the realisation that people with chronic illnesses have to do this all the time.—20, inner regional, diagnosed less than a year ago.

The impact of increased online social activities was either seen to be a “bit exhausting” (*n* = 1), or as a way to participate in newly virtual social activities that were previously inaccessible (*n* = 1).

Stress was a common issue (*n* = 19) and was often a trigger for flare-ups or increasing pain. Stressors included COVID-19, work, accessing care, and managing endometriosis.

Treatment of endometriosis is hard enough and wait times are crazy long … since this virus getting treatment or even just seeing the gyno never less surgery not only long but the mental stress of waiting even longer is [phenomenal].—34, major city, diagnosed 1–5 years ago.

The management of mental health was difficult (*n* = 12), but isolation allowed new time and space to rest (*n* = 1). Four participants also used the time to reflect and come to terms with endometriosis and its effects; for one participant the time in isolation helped her to decide she “wasn't ready at 39 for a third surgery,” whereas another decided to “prioritise integrating care and treatment of endo into [their] daily life.”

For some (*n* = 4), changes due to COVID-19 did not affect their lives as lockdown restrictions characterized their everyday with a chronic illness.

## Discussion

The impact of coronavirus-related policy changes on individuals with endometriosis has been considerable, particularly with regard to diminished access to health care and symptom management techniques. Our study offers novel insights into how communications from the medical community are received by people with chronic health conditions and gives voice to suggestions of service delivery from a patient community with highly variable needs and a complex relationship with institutional medicine.

Unclear communication around health service changes was a key issue that resulted in feelings of neglect among participants. In Australia, surgeries are typically categorized as “elective” or “non-elective” and as category 1 (to be completed in 30 days), 2 (to be completed in 90 days), and 3 (to be completed in 1 year).^15^ Endometriosis surgeries are generally classed as elective category 3 surgeries^15^ (*i.e*., considered “non-essential” from the perspective of reduced medical services), yet this was in conflict with participants' views that surgery was essential to their well-being. Communication around the cancellation of elective procedures was interpreted as dismissal of their pain. This illustrates the need for clear empathic messaging from authorities and the medical community to reduce feelings of confusion, frustration, dismissal, and neglect, which are common for those with endometriosis.^16–19^ Engaging appropriately with patient groups can minimize distress and foster a more supportive environment for patients.

It is well known that endometriosis patients self-manage pain.^7^ This was further emphasized during pandemic-related restrictions, with respondents finding success with self-management techniques at home. Our findings confirm that chronic symptoms are often exacerbated in the workplace,^20^ yet many employees, in particular women,^21^ do not feel comfortable requesting modifications to their environment to alleviate symptoms.^22^ The strategies used while working from home—heat packs, comfortable clothing, lying down, and working at their own pace during office hours—were key factors that resulted in fewer symptom flare-ups and improved productivity.

This speaks to the “hidden benefits” of being able to work in the comfort of one's own home.^23^ This illustrates that postpandemic, to better support people with chronic pain (such as endometriosis) employers should consider offering the option to work from home, as well as making workplaces more conducive to self-management techniques. This would not only improve the control of symptoms and ultimately improve health-related quality of life for workers, but would also reduce absenteeism and presenteeism.

Furthermore, although telehealth services were generally thought to be inferior to face-to-face consultations, for those living rurally or who had mobility difficulties, telehealth reduced barriers to medical and allied health services. In addition, participants found it useful for repeat prescriptions, as it reduced the need to travel and, therefore, discomfort. This technology should continue beyond the pandemic to improve access for those who want it.

A strength of this study is the large sample size. The main limitation is the online anonymous survey format, which precluded the recursive process that strengthens qualitative data collection. Furthermore, recruiting from online support communities may have overselected those with more urgent needs or more engagement with endometriosis care, and thus our sample may not be representative.^24^ The sampling strategy may also explain the predominance of Australian-born English-speaking tertiary-educated participants.

This study provides insight into changes in health care delivery that have been both beneficial and harmful to those with endometriosis and, by extension, potentially, people with other chronic conditions. There were some obvious negative impacts of the COVID-19 pandemic, such as reduced health care access and confusing communication from policy makers and health care professionals. However, positive impacts were seen in pain management due to working from home, and there were mixed impacts from changed services (*e.g*., telehealth).

Many of the negative impacts of pandemic-related changes (*e.g*., canceled medical appointments) will resolve postpandemic as services resume; however, the positive impacts provide evidence for systems (*e.g*., flexible work-from-home arrangements and telehealth) that can continue to be utilized postpandemic to improve the lives of people with endometriosis. Also highlighted in this study is the importance of mindful communication of health care policies from policy makers so as to reduce harm to patient well-being.

## Abbreviations Used

IQR: interquartile range
IVF: *in vitro* fertilisation
TCM: traditional Chinese medicine
